# Extended Line Defect Graphene Modified by the Adsorption of Mn Atoms and Its Properties of Adsorbing CH_4_

**DOI:** 10.3390/nano12040697

**Published:** 2022-02-19

**Authors:** Chenxiaoyu Zhang, Shaobin Yang, Xu Zhang, Yingkai Xia, Jiarui Li

**Affiliations:** College of Material Science and Engineering, Liaoning Technical University, Fuxin 123000, China; ZCXY202201@163.com (C.Z.); zhangxu220120@163.com (X.Z.); xiayingkai200719@126.com (Y.X.); LJR220128@163.com (J.L.)

**Keywords:** first principles, graphene, extended line defect, CH_4_ adsorption, Mn modification

## Abstract

Extended line defect (ELD) graphene is a two-dimensional (2D) topologically defective graphene with alternate octagonal and quadrilateral carbon rings as basic defective units. This paper reports on the CH_4_ adsorption properties of ELD graphene according to the first principles of density functional theory (DFT). The effects on the CH_4_ adsorption of ELD graphene when modified by a single Mn atom or two Mn atoms were investigated, respectively. An ELD-42C graphene configuration consisting of 42 C atoms was first constructed. Then, the ELD-42C graphene configuration was used as a substrate, and a Mn-ELD-42C graphene configuration was obtained by modifying it with a single Mn atom. The results showed that the most stable adsorption site for Mn atoms was above the quadrilateral carbon ring. This Mn-ELD-42C graphene configuration could only stably adsorb up to 30 CH_4_ molecules on each side, with an average adsorption energy of −0.867 eV/CH_4_ and an adsorption capacity of 46.25 wt%. Three 2Mn-ELD-42C graphene configurations were then obtained by modifying the ELD-42C graphene substrate with two Mn atoms. When the two Mn atoms were located on either side of a 2Mn-ELD-42C graphene configuration and above the two octagonal carbon rings adjacent to the same quadrilateral carbon ring, it was able to adsorb up to 40 CH_4_ molecules on each side, with an average adsorption energy of −0.862 eV/CH_4_ and a CH_4_ adsorption capacity of 51.09 wt%.

## 1. Introduction

CH_4_ is abundant in nature and has a higher energy density than fossil fuels such as petroleum and coal. It is also a relatively clean fuel, with the lowest rate of CO_2_ emissions of all carbonaceous fuels [1,2]. It has therefore been widely recognized as a transitional resource until alternatives to oil and coal can be found and developed on a large enough scale [3,4]. As a result, studies on CH_4_ adsorption and its storage have important practical significance for energy development and use as well as environmental protection.

Graphene is a new kind of two-dimensional (2D) honeycomb-shaped nanomaterial characterized by good mechanical properties, good hydrogen storage and a high sensitivity to as well as adsorption potential for certain types of gases [5]. Zhao et al. [6] found that pristine graphene has a weak adsorption capacity for CH_4_, with an average adsorption energy of −0.227 eV/CH_4_. Ghanbari et al. [7] found that the adsorption energy could be improved to −0.166 eV when the graphene was modified with Ag atoms (Ag-G). This implies that physical absorption occurs between Ag atoms and graphene. Xu et al. [8] used Ti atoms to modify graphene and found that the modified graphene was most stable when the Ti atoms were located above the top carbon rings. This had an average adsorption energy of −0.298 eV/CH_4_. The United States Department of Energy (DOE) established a CH_4_ storage objective for vehicles in 2012, where the goal was to have a CH_4_ adsorption capacity of above 50 wt% under standard conditions [9]. Unfortunately, the majority of CH_4_ storage materials still fail to meet this requirement.

Pristine graphene is composed of a single layer of carbon atoms in the form of sp2 hybrid orbits and has a perfect hexagonal carbon ring structure [10]. During its growth, graphene typically acquires defects such as monovacancy, divacancy, Stone–Wales (SW), and topological lines [11]. These defects have been proven to be conducive to the adsorption of CH_4_. Xiong et al. [12] constructed an extended line defect (ELD) graphene periodically embedded with quadrilateral and octagonal carbon rings by means of surface synthesis. ELD graphene is a type of semiconductor with a unique carbon ring structure and special chemical, electronic and mechanical properties, with a reduced band gap [13]. A large number of studies have shown that the CH_4_ adsorption properties of graphene can be further improved by doping atoms to introduce additional structural modifications. Existing studies on transition metal (TM)-modified ELD graphene have concentrated on the magnetic and electronic properties of the configurations after modification. Cheng et al. [14] found that a single TM atom is preferentially adsorbed at defect sites in ELD graphene where there is high chemical activity. When modified by TM atoms, an ELD graphene substrate displays magnetism and spin polarization. Yu et al. [15] investigated the 558-type ELD, which is composed of a periodic repetition of one octagonal and two pentagonal rings, embedded in the hexagonal lattice of a graphene sheet. They found that the magnetism of the TM atom depends on the adsorption sites and the type of the adatoms, which can be obtained by analyzing the underlying hybridization mechanism between 3d orbitals of the TM atom and the electronic states of the ELD. Guan et al. [16] calculated defective graphene nanoribbon and TM adsorption on a line-defective embedded graphene sheet. The results show that TM atom adsorption on graphene can introduce magnetism and spin polarization, which is at the ferromagnetic ground state and shows different electronic properties according to different metals.

Manganese (Mn) is a transition metal, with reserves of about 570 million tons worldwide, that can be made by aluminothermic reduction of soft manganese ore [17,18]. Manganese is easily oxidized to manganese dioxide [19]. Manganese dioxide is an excellent adsorbent material due to its large specific surface area and strong electrochemical properties [20]. Manganese is located in the fourth period of the chemical periodic table in Group VIIB. The valence electronic configuration of manganese atom is 3d^5^4s^2^, with more electrons and empty orbits at the 3d energy level, which helps to enhance chemical bonding between manganese atoms and other molecules, allowing manganese atoms to adsorb gaseous molecules more efficiently [21,22]. In the laboratory, potassium permanganate or manganese dioxide is used as a catalyst to prepare graphene, resulting in the produced graphene containing a certain number of Mn atoms in its structure [23,24].

This paper presents an analysis of the adsorption properties of ELD graphene for CH_4_, working from first principles [25]. It looks at the CH_4_ adsorption properties of ELD graphene modified with a single Mn atom or two Mn atoms and calculates the resulting CH_4_ adsorption capacity. This study offers theoretical support for the preparation and industrial application of new CH_4_ storage materials.

## 2. Calculation Methods and Models

This paper is based on a first-principles pseudopotential plane-wave (PSPW) method that comes from density functional theory (DFT) [26,27]. DFT calculations have been applied successfully to analyze the defective carbon-based graphene-like systems containing the same type of defects as in this paper, such as carbon-based fullerene-like sulfocarbide [28], fullerene-like phosphorus carbide [29] and graphene-like model systems based on coronene and corannulene molecules [30]. The goal of the study was to investigate the CH_4_ adsorption properties of ELD graphene as well as ELD graphene when modified by a single Mn atom or two Mn atoms at an atomic level by using the Cambridge Sequential Total Energy Package (CASTEP) module in the Materials Studio software [31]. Perdew–Burke–Ernzerhof (PBE) and generalized gradient approximation (GGA) functionals were selected for the calculations [32], and the interaction between electrons and ions was approximately calculated using OTFG ultrasoft. If the adsorption energy calculated by the GGA functional is weak, the adsorption energy can be corrected by means of a DFT dispersion correction (van der Waals) functional (namely DFT-D) [33]. The convergence criteria to optimize the calculations for atoms within the objects of study were set as follows: a maximum stress of 0.05 eV/Å; a maximum displacement of 0.002 Å; a convergence energy of 2.0 × 10^−5^ eV/atom; and a self-consistent field (SCF tolerance) convergence threshold of 2.0 × 10^−6^ eV/atom. To ensure the calculation accuracy and reduce the calculation cost, the truncation energy was set at 450 eV, the K-point sampling was set at 5 × 5 × 1, and the objects of study were integrated in a Brillouin zone with Monkhorst–Pack grids [34,35]. The periodic boundary conditions to be met for the calculation of ELD graphene unit cells and the vacuum layer were set at 30 Å to avoid mutual interference between layers.

**Figure 1:**
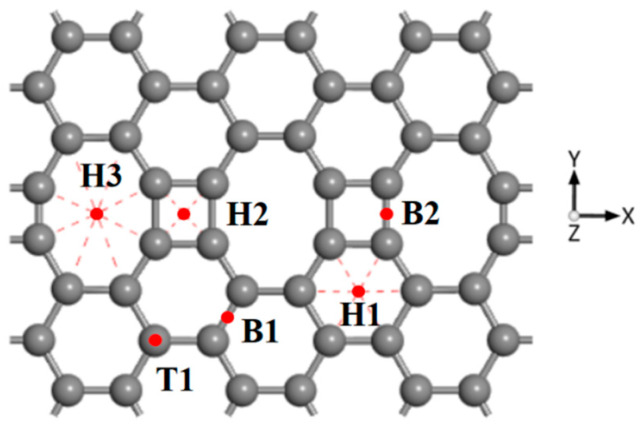
ELD-42C graphene configuration after structural optimization (the gray balls represent carbon atoms). H1, H2 and H3 indicate the hole sites; T1 stands for the top site; and B1 as well as B2 refer to the bridge sites.

An ELD-42C graphene configuration consisting of 42 C atoms was constructed using graphene unit cells, with a main body that was composed of continuous quadrilateral, hexagonal and octagonal carbon rings. Its geometrical configuration after structural optimization is shown in Figure 1. The length of all the C-C bonds that constitute pristine graphene is 1.42 Å. According to an analysis of the ELD-42C graphene configuration after optimization, the lengths of the three C-C bonds that constituted the octagonal carbon rings were 1.48 Å, 1.39 Å and 1.41 Å, respectively. For the two C-C bonds that constituted the quadrilateral carbon rings, the lengths were 1.39 Å and 1.47 Å, respectively. In comparison to pristine graphene, some of the C-C bonds (1.39 Å and 1.41 Å) of the ELD-42C graphene configuration were slightly compressed, and some (1.48 Å and 1.47 Å) were slightly stretched. This is consistent with the experimental results in Liu et al. [36] and Zhao et al. [37] and coincides closely with the simulation results in Ding et al. [38]. This confirms the validity of the ELD-42C graphene configuration design.

For the *iMn-ELD*-42*C* graphene configurations obtained after modification by the *Mn* atoms, the binding energy, $E_{b_{iMn}}$, and average binding energy, $\bar{E_{b}{}_{{}_{iMn}}}$, of the *Mn* atoms can be defined as follows:

(1) $$E_{b_{iMn}}=E_{iMn-ELD-42C}-(E_{ELD-42C}+E_{iMn})$$

(2) $$\bar{E_{b_{iMn}}}=[E_{iMn-ELD-42C}-E_{ELD-42C}-E_{iMn}]/i$$

where *i* indicates the number of *Mn* atoms for modification; $E_{iMn-ELD-42C}$ refers to the total energy of the *iMn*-*ELD*-42*C* graphene configurations; $E_{ELD-42C}$ stands for the total energy of the *ELD*-42*C* graphene configurations; and $E_{iMn}$ represents the total energy of the *i*-free *Mn* atom(s).

For the *CH*_4_ molecules in the *jCH*_4_↔*iMn-ELD*-42*C* graphene adsorption configurations, the binding energy, $E_{ad}$, average binding energy, $\bar{E_{ad}}$, and PBW by percentage of weight can be defined as follows:

(3) $$E_{ad}=E_{jCH_{4}↔iMn-ELD-42C}-E_{(j-1)CH_{4}↔iMn-ELD-42C}-E_{CH_{4}}$$

(4) $$\bar{E_{ad}}=[E_{jCH_{4}↔iMn-ELD-42C}-E_{iMn-ELD-42C}-jE_{CH_{4}}]/j$$

where *j* indicates the number of *CH*_4_ molecule(s) adsorbed; $E_{jCH_{4}↔iMn-ELD-42C}$ refers to the total energy of the *jCH*_4_↔*iMn-ELD*-42*C* graphene adsorption configurations; $E_{(j-1)CH_{4}↔iMn-ELD-42C}$ stands for the total energy of the (*j* − 1)*CH*_4_↔*iMn-ELD*-42*C* graphene adsorption configurations; $jE_{CH_{4}}$ represents the total energy of the *j*-free *CH*_4_ molecule(s); and $M_{r(CH_{4})}$, $M_{r(Mn)}$ and $M_{r(ELD)}$ represent the weight of each *CH*_4_ molecule, each Mn atom and the ELD system, respectively.

## 3. Results and Discussion

### 3.1. CH_4_ Adsorption in the ELD-42C Graphene Configuration

**Figure 2:**
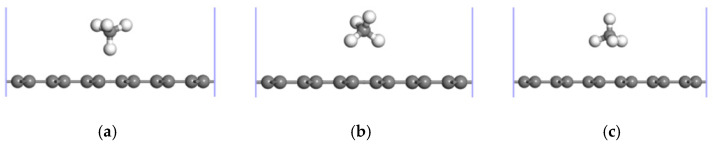
Three adsorption forms of CH_4_ molecules in relation to the ELD-42C graphene configuration. (**a**–**c**) indicate that 1, 2 or 3 H atom(s) in the CH_4_ molecule are oriented to the plane of the ELD-42C graphene configuration, respectively.

Six typical CH_4_ adsorption sites were selected to be studied on account of the symmetry of the geometric structure of the ELD-42C graphene configuration, as shown in Figure 1. H indicates a hole in a carbon ring, with H1, H2 and H3 representing the centroid sites of the hexagonal, quadrilateral and octagonal carbon rings, respectively. T1 stands for the top site of a C atom and B refers to the bridge site of a C-C bond, with B1 indicating the bridge sites in the hexagonal carbon rings and B2 representing the bridge sites in the defective rings (i.e., the quadrilateral and octagonal carbon rings). There were three adsorption forms for CH_4_ molecules adsorbed on the ELD-42C graphene configuration according to the orientations of the four H atoms of the CH_4_ molecules relative to the plane of the ELD-42C graphene configuration, as shown in Figure 2. Adsorption properties are affected by the adsorption sites for CH_4_ molecules on the graphene, not the orientation of H atoms in the CH_4_ molecules [39]. Therefore, the CH_4_ adsorption form with three H atoms orientated to the plane of the ELD-42C graphene configuration (Figure 2c) was selected to study the adsorption properties at different sites. The CH_4_ adsorption energy, $E_{ad}$; vertical distance, d, between the C atoms in the CH_4_ molecules and the plane of the ELD-42C graphene configuration; and bond angle, ∠H-C-H, between the C atoms and the H atoms in the CH_4_ molecules were calculated for a CH_4_ molecule adsorbed at the six typical adsorption sites of the ELD-42C graphene configuration. The results are shown in Table 1.

**Figure 3:**
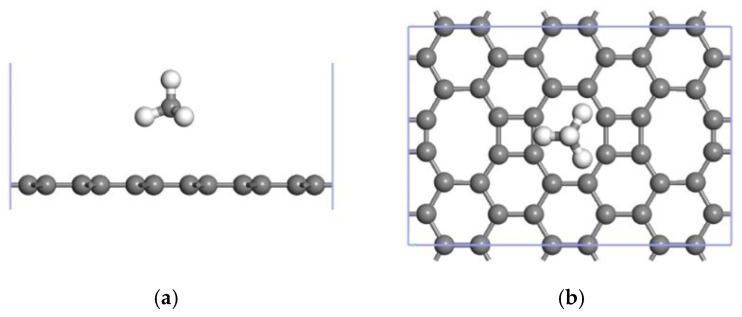
CH_4_↔ELD-42C graphene adsorption configuration with 1 CH_4_ molecule being adsorbed at H3 (gray balls represent carbon atoms, and white balls represent hydrogen atoms). (**a**) Front view; (**b**) top view.

Table 1 shows the adsorption energy released, $E_{ad}$, after one CH_4_ molecule was adsorbed at the six adsorption sites in the ELD-42C graphene configuration. The larger its absolute value, the more energy released and the more stable the corresponding CH_4_↔ ELD-42C graphene adsorption configuration. It can be seen that the largest absolute value (−0.847 eV) was at H3, indicating that the ELD-42C graphene configuration was at its most stable at H3 when adsorbing CH_4_ molecules, compared with the other five adsorption sites. Thus, the CH_4_ molecules tended to stay above the octagonal carbon ring, as shown in Figure 3. For free CH_4_ molecules, the length of bonds between the C and H atoms is 1.110 Å, and the bond angle is 109.381°. According to [40], the adsorption height of gas molecules is about three times their bond length, so the adsorption height of the CH_4_ molecules was preset as 3.28 Å. After structural optimization, the vertical distance, d, between the CH_4_ molecules at the six adsorption sites and the plane of the ELD-42C graphene configuration was analyzed. It was found that the difference between the stable adsorption height and the preset adsorption height was between 0.002 Å and 0.199 Å, indicating that the preset adsorption height was reasonable. The bond angle between the C and H atoms was close to that of CH_4_ molecules adsorbed by the ELD-42C graphene configuration in their free state [41,42], so the adsorption for CH_4_ on the ELD-42C graphene configuration was physical.

**Figure 4:**
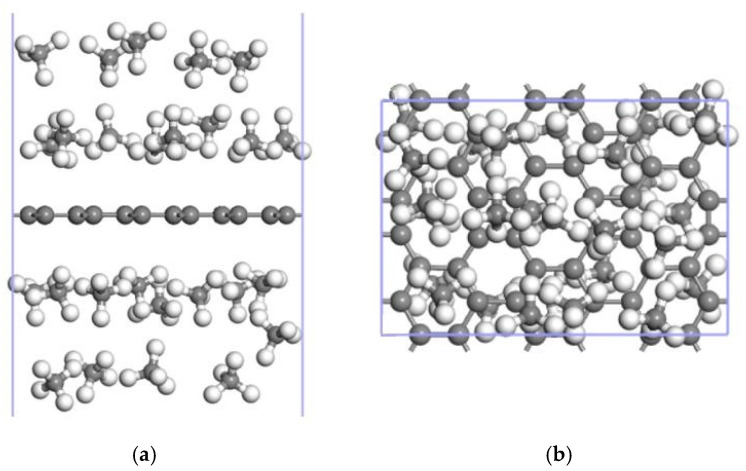
8CH_4_↔ELD-42C graphene adsorption configuration (gray balls represent carbon atoms, and white balls represent hydrogen carbons). (**a**) Front view; (**b**) top view.

CH_4_ molecules were preferentially adsorbed by the ELD-42C graphene configuration at H3. They were then adsorbed at T1 after being fully adsorbed at H3. The ELD-42C graphene configuration could stably adsorb 26 CH_4_ molecules at most on each side, with an average adsorption energy of −0.842 eV/CH_4_ and an adsorption configuration similar to the one shown in Figure 4. According to the technical standards for natural gas adsorption systems issued by the DOE [43], the adsorption capacity of CH_4_ storage materials should not be less than 50 wt%. For 26CH_4_↔ELD-42C graphene adsorption configurations, the CH_4_ adsorption capacity was 45.26 wt%, which is lower than the technical standard; therefore, the ELD-42C graphene configuration was still not suitable for practical applications.

### 3.2. CH_4_ Adsorption in the Mn-ELD-42C Graphene Configuration

#### 3.2.1. Modification of the ELD-42C Graphene Configuration by a Single Mn Atom

Jia et al. [2] found that pristine graphene doped with heteroatoms or pristine graphene with structural defects is significantly better able to adsorb gas molecules. Xu et al. [44] believed that the adsorption properties of graphene for gas molecules can be best improved by modifying graphene with alkali metals, alkaline earth metals and TM. Mn is an important TM element that is widely distributed throughout the Earth’s crust. Its valence electron configuration is 3d^5^4s^2^, and chemical bonds can be easily formed between Mn and carbon atoms [45,46,47]. Due to its weak adsorption properties for CH_4_ molecules, the ELD-42C graphene configuration was modified with TM Mn atoms to construct iMn-ELD-42C graphene configurations (where *i* indicates the number of Mn atoms, *i* = 1, 2). Their adsorption properties for CH_4_ were then studied.

When the ELD-42C graphene configuration was modified by a single Mn atom, there were six optional adsorption sites for the Mn atoms: the hole sites, H1, H2 and H3; the bridge sites, B1 and B2; and the top site, T1 (see Figure 1). A single Mn atom, respectively placed at T1, B1 and B2 during the construction of the Mn-ELD-42C configurations, always moved to the top of the adjacent carbon ring under the action of the chemical bonds as the structure was optimized. This is in line with the optimal adsorption site of TM atoms determined by Zhao et al. [6] and Liu et al. [48]. The adsorption characteristics of the Mn atoms at H1, H2 and H3 were calculated, and the results are given in Table 2.

**Figure 5:**
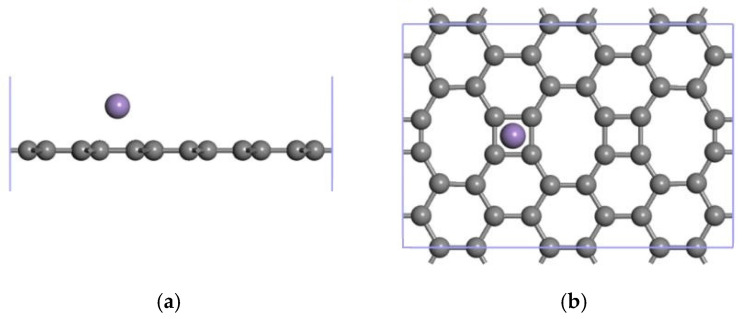
The most stable Mn-ELD-42C graphene configuration was obtained by modifying the hole site H2 with a single Mn atom (gray balls represent carbon atoms, and purple balls represent Mn atoms). (**a**) Front view; (**b**) top view.

In the table, $E_{b_{Mn}}(eV)$ indicates the binding energy of a single Mn atom; BL1, BL2, BL3 and BL4, respectively, refer to the length of the bonds between the Mn atoms and C atoms; and Δρ(e) represents the charge transfer between the Mn atoms and the ELD-42C graphene configuration. It can be seen from Table 2 that the binding energy of a single Mn atom was different at H1, H2 and H3. At H2 it was −3.453 eV. This was the largest absolute value out of the three hole sites. The binding energy of a single Mn atom at H1 was −2.922 eV, which was the smallest absolute value. These results indicated that Mn atoms adsorbed above H2 were the most stable, while Mn atoms adsorbed above H1 were the least stable. During modification, four Mn-C chemical bonds were formed between the Mn atoms and the four carbon atoms at H2, with lengths of 2.967 Å, 2.974 Å, 2.974 Å and 2.981 Å, respectively. This suggests that most of the Mn atoms were adsorbed on the central axis of H2. The charge transferred from the Mn atoms to the ELD-42C graphene configuration was 0.33 e at H3 and 0.29 e at H1. Therefore, the interaction between the ELD-42C graphene configuration and Mn atoms adsorbed at H1 and H3 was weaker than it was at H2. The most stable Mn-ELD-42C graphene system configuration (Figure 5) was therefore obtained via structural optimization after it had been modified by a single Mn atom at H2.

**Figure 6:**
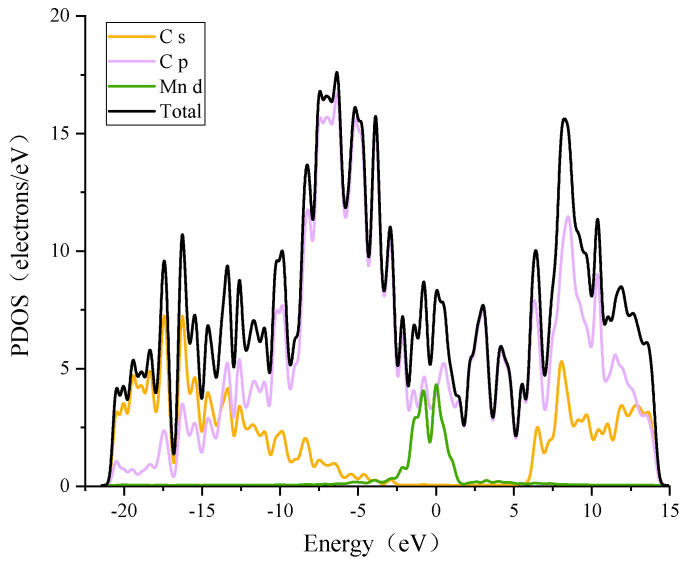
PDOS diagram of the Mn-ELD-42C graphene system (partial).

The Mulliken layout of the Mn-ELD-42C graphene configuration before and after adsorbing a single CH_4_ molecule was analyzed. It was found that the charge transferred from the Mn atoms to the ELD-42C graphene configuration was 0.69 e, indicating a strong electrostatic effect between the two. The partial density of states (PDOS) for the Mn-ELD-42C graphene configuration is shown in Figure 6 (partial). There were resonance peaks between the d orbit of the Mn atoms and the *p* orbit of the C atoms within the range of −1.958 to −1.381 eV, confirming that there was interaction between the two orbits. As a result, the valence band of the Mn-ELD-42C graphene configuration largely derives from the interaction between the d orbit of the Mn atoms and the *p* orbit of the C atoms. This is similar to the results obtained by Wu et al. [49] and Zhao et al. [37], who modified graphene substrates by using TM atoms as a doping agent. As extra electrons were provided to the ELD-42C graphene configuration by the Mn atoms, the overall conduction band of the configuration moved to the Fermi level, where the conduction band intersected with the valence band and endowed the Mn-ELD-42C graphene configuration with typical metallic-phase characteristics.

#### 3.2.2. Adsorption of CH_4_ by the Mn-ELD-42C Graphene Configuration

**Figure 7:**
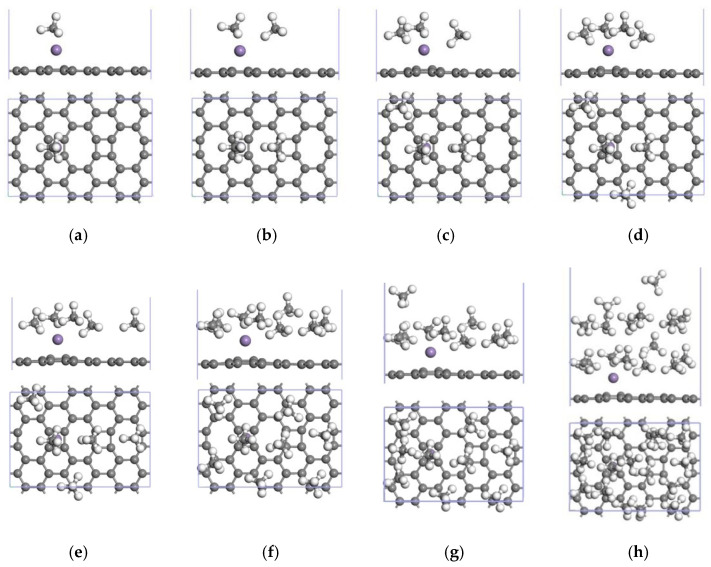
jCH_4_↔Mn-ELD-42C adsorption configurations (*j* = 1, 2, …, 16). Figures (**a**–**h**) respectively demonstrate the Mn-ELD-42C graphene configurations for 1–16 CH_4_ molecule(s) adsorbed on one side.

DFT was used to study the CH_4_ adsorption capacity of the Mn-ELD-42C graphene configuration by adding CH_4_ molecules to one side. A stable CH_4_↔Mn-ELD-42C adsorption configuration (Figure 7a) was obtained after the Mn-ELD-42C graphene configuration with the first adsorbed CH_4_ molecule had been optimized. The first CH_4_ molecule was located above the Mn atom, proving that this was where the adsorption energy was the largest. The adsorption energy of this configuration was −1.717 eV, which is larger than that of the ELD-42C graphene configuration for CH_4_ molecules (−0.847 eV), of Li-modified carbon nanotubes for CH_4_ (−0.464 eV) [50] and of Pt-modified graphene for CH_4_ (−0.488 eV) [51]. Apparently, modifying the ELD-42C graphene configuration with a single Mn atom improved its adsorption properties for CH_4_ molecules. A 2CH_4_↔Mn-ELD-42C adsorption configuration was obtained after a second CH_4_ molecule had been added (Figure 7b). Here, both the first and the second CH_4_ molecules were located above the Mn atoms and close to the Mn-ELD-42C graphene configuration. The combined action of the mutual repulsion of the CH_4_ molecules and their adsorption by the Mn-ELD-42C graphene configuration enabled a third CH_4_ molecule to be adsorbed above the hexagonal carbon ring that was close to the Mn atoms (Figure 7c). A fourth CH_4_ molecule was adsorbed at T1 above the C atoms (Figure 7d), and a fifth above the octagonal carbon ring (Figure 7e). Limited by the adsorption space, the repulsive force between the molecules gradually increased as more CH_4_ molecules were adsorbed. The adsorption configuration began to arc when an eighth molecule was adsorbed (Figure 7f), and there was a stratification phenomenon when the ninth molecule was adsorbed (Figure 7g). Due to their layered adsorption, the distances between the 9th–16th CH_4_ molecules and the Mn atom became larger, and the adsorption energy was reduced. The 16th CH_4_ molecule was nowhere near the Mn atom and was the most distant from the Mn-ELD-42C graphene configuration. It also had the lowest adsorption energy (−0.755 eV). When a 17th CH_4_ molecule was placed on one side of the configuration, the calculated adsorption energy became positive, indicating that the gas molecule had not been adsorbed. This proved that the Mn-ELD-42C graphene configuration could only stably adsorb up to 16 CH_4_ molecules on each side. The geometrical configuration is shown in Figure 7h.

Table 3 shows the average adsorption energy, $\bar{E_{ad}}$, and the adsorption energy, $E_{ad}$, of the jCH_4_↔Mn-ELD-42C↔jCH_4_ adsorption configuration for CH_4_ adsorption on one side and both sides; the distance, $d_{CH_{4}-S}$, between the CH_4_ molecules and the plane of the Mn-ELD-42C graphene configuration; the distance, $d_{CH_{4}-Mn}$, between the CH_4_ molecules and the Mn atoms; and the adsorption capacity (PBW) of the Mn-ELD-42C graphene configuration for CH_4_ in the jCH_4_↔Mn-ELD-42C↔jCH_4_ adsorption configuration. Analysis of these data reveals that the absolute value of the average adsorption energy, $\bar{E_{ad}}$, of the CH_4_ gradually decreased as j, the number of CH_4_ molecules adsorbed, increased. The adsorption energy, $E_{ad}$, of 16 CH_4_ molecules adsorbed on one side was compared. When the gas molecules were not stratified (the first–eighth molecules), the first, third, and seventh CH_4_ molecules presented a higher adsorption energy than the other CH_4_ molecules because their adsorption sites were close to Mn atoms and their distance from the plane of the graphene configuration was relatively small. When the gas molecules were stratified (the ninth–sixteenth molecules), the interaction between the CH_4_ and Mn steadily decreased as the distance between them increased, leading to reduced adsorption properties.

The above results indicate that the adsorption properties of a Mn-ELD-42C graphene configuration are affected by Mn atom modification and that this can play an important role in CH_4_ adsorption. The adsorption energy was also affected by the distance between the CH_4_ molecules and the plane of the graphene configuration. The adsorption distance, $d_{CH_{4}-S}$, between the 16th CH_4_ molecule and the Mn-ELD-42C graphene configuration was 11.550 Å, which was the largest out of the 16 CH_4_ molecules. At this point, both the average adsorption energy, $\bar{E_{ad}}$, and the adsorption energy, $E_{ad}$, of the configuration were at their lowest (−0.897 eV/CH_4_ and −0.755 eV, respectively).

**Figure 8:**
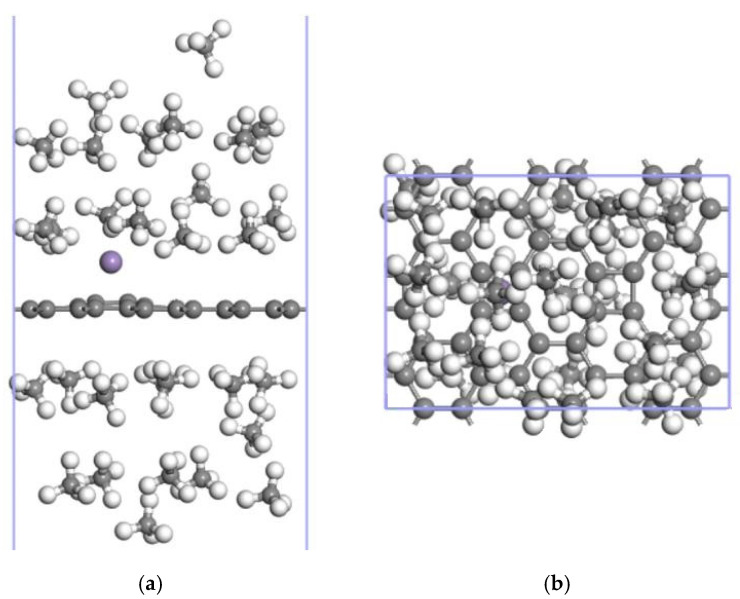
16CH_4_↔Mn-ELD-42C↔14CH_4_ adsorption configuration with 16 and 14 CH_4_ molecules, respectively, adsorbed on each side. (**a**) Front view; (**b**) top view (gray balls represent carbon atoms, white balls stand for hydrogen atoms and purple balls represent Mn atoms).

Up to 16 CH_4_ molecules could be stably adsorbed on one side of the Mn-ELD-42C graphene configuration, with an average adsorption energy of −0.897 eV/CH_4_. On this basis, it can be calculated that the Mn-ELD-42C graphene configuration is able to stably adsorb up to 14 CH_4_ molecules on the other side, making a total of 30 CH_4_ molecules overall (Figure 8), with an average adsorption energy of −0.867 eV/CH_4_ and an adsorption capacity of 46.25 wt%. This is much closer to the proposed DOE standard (50 wt%) [9]. The adsorption capacity of the Mn-ELD-42C graphene configuration was 1.02 times that of the basic ELD-42C graphene configuration (45.26 wt%). This makes it clear that the adsorption capacity for CH_4_ molecules can be effectively improved by the modification of Mn atoms.

Table 4 gives the Mulliken layout of the Mn-ELD-42C graphene configuration before and after adsorbing one CH_4_ molecule, where H_1_, H_2_, H_3_ and H_4_ stand for the H atoms and C represents the C atom of the CH_4_ molecule. For the CH_4_ molecule adsorbed above the Mn atom, H_1_, H_2_ and H_3_ faced the plane of the Mn-ELD-42C graphene configuration, while H4 faced away from it (Figure 6a). The charge for H_4_ was 0.27 e before the CH_4_ molecule was adsorbed and 0.36 e after the CH_4_ molecule was adsorbed, with 0.09 e of charge having been lost. For free CH_4_ molecules, the C atom is negatively charged, and the four peripheral H atoms are positively charged. This results in a strong repulsive force between CH_4_ molecules, making it difficult for multiple CH_4_ molecules to gather at the same adsorption site. For the Mn-ELD-42C graphene configuration, the ELD-42C graphene substrate was negatively charged, allowing the positively charged CH_4_ molecules on the outer surface to be adsorbed more easily via electrostatic interaction. In the CH_4_ molecules adsorbed on the Mn-ELD-42C graphene configuration, both H_1_ and H_2_ lost their partial positive charge because they received equal numbers of electrons. This reduced the surface area of the positively charged CH_4_ molecule, weakening the repulsive force between the CH_4_ molecules. In addition, before and after a single CH_4_ molecule had been adsorbed by the Mn-ELD-42C graphene configuration, a relatively large charge transfer occurred with the Mn atoms, with 0.29 e of charge being lost. When CH_4_ molecules were adsorbed, the electrons of the Mn atoms were transferred to the CH_4_ molecules; therefore, a strong Coulomb force was produced between the two, creating favorable conditions for CH_4_ adsorption.

**Figure 9:**
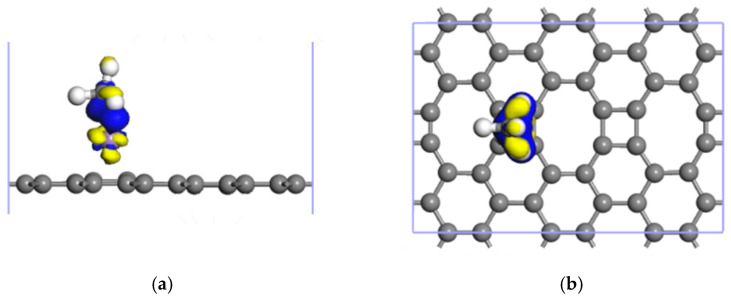
Charge density difference for the CH_4_↔Mn-ELD-42C adsorption configuration. (**a**) Front view; (**b**) top view.

Figure 9 illustrates the charge density difference for the CH_4_↔Mn-ELD-42C adsorption configuration. This directly reveals the charge transfer between the Mn atoms and CH_4_ molecules. The blue elements are the electron gain zone, where the CH_4_ molecules obtained electrons, and the yellow elements are the electron loss zone, where the Mn atoms lost electrons. As the large charge transfer between the Mn atoms and CH_4_ molecules produced a Coulomb force between them, the Mn atoms had a significant effect on CH_4_ adsorption. This is consistent with the analysis of the Mulliken layout in Table 4.

**Figure 10:**
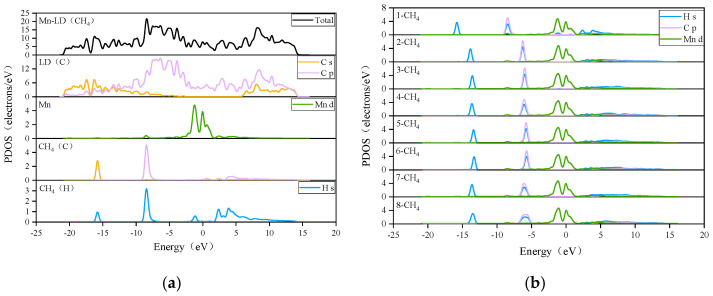
PDOS of the CH_4_ molecules in the jCH_4_↔Mn-ELD-42C adsorption configuration. (**a**) For the CH_4_↔Mn-ELD-42C adsorption configuration; (**b**) for the (1–8)CH_4_↔Mn-ELD-42C adsorption configurations.

The interaction between the Mn-ELD-42C graphene configuration and CH_4_ molecules was also analyzed in terms of the PDOS of the CH_4_ molecules. Figure 10a shows the PDOS of the CH_4_↔Mn-ELD-42C adsorption configuration after adsorbing a single CH_4_ molecule. The density of states (DOS) peak of the Mn atoms increased from 4.031 eV (before adsorption, as shown in Figure 6) to 4.763 eV (after adsorption), and the energy range enlarged from (−4.107, 1.547 eV) before adsorption to (−4.341, 1.637 eV) after adsorption. As a consequence, the CH_4_ adsorption enhanced the interaction between the Mn atoms and the Mn-ELD-42C graphene configuration, which is in accord with the analysis of the Mulliken layout in Table 4. After a single CH_4_ molecule had been adsorbed, the DOS valence band peak of the CH_4_↔Mn-ELD-42C adsorption configuration improved because of the hybridization between the 3d orbit of the Mn atoms and the 1s orbit of the H atoms. The DOS of the C atom in the Mn-ELD-42C graphene configuration also changed slightly.

Figure 10b was used to analyze the interaction between the d orbit of the Mn atoms, the s orbit of the H atoms and the *p* orbit of the C atom on the eight unstratified CH_4_ molecules adsorbed on one side of the Mn-ELD-42C graphene configuration. It can be seen that the s orbit of the H atoms of the first CH_4_ molecule overlapped with the 3d orbit of the Mn atoms near −16.0 eV and −8.0 eV. This suggests that there is an interaction between the first CH_4_ molecule and the Mn atoms. Compared with the first CH_4_ molecule, the 1s orbit of the second CH_4_ molecule had shifted to the right, indicating that the interaction between the second CH_4_ molecule and the Mn atoms had weakened, making the adsorption energy of the second CH_4_ molecule smaller than that of the first CH_4_ molecule. The displacement of the PDOS peak for the CH_4_ molecules correlated with changes in the adsorption energy, with the PDOS peak moving to the left when the adsorption energy increased and to the right when the adsorption energy reduced. As the number of CH_4_ molecules adsorbed increased, the PDOS peak reduced and moved to the right, showing that the interaction between the CH_4_ molecules and Mn atoms was gradually weakening. In the interval [−6.989, −4.893 eV], the PDOS peak of the eighth CH_4_ molecule was significantly lower than that of the other seven CH_4_ molecules, indicating that the interaction between the 1s orbit of the H atoms of the eight CH_4_ molecule and the 3d orbit of the Mn atoms was the weakest. This is consistent with the gradual decrease in the average adsorption energy when the CH_4_ molecules were adsorbed by the Mn-ELD-42C graphene configuration.

### 3.3. CH_4_ Adsorption in the 2Mn-ELD-42C Graphene Configuration

#### 3.3.1. Modification of the ELD-42C Graphene Configuration by Two Mn Atoms

**Figure 11:**
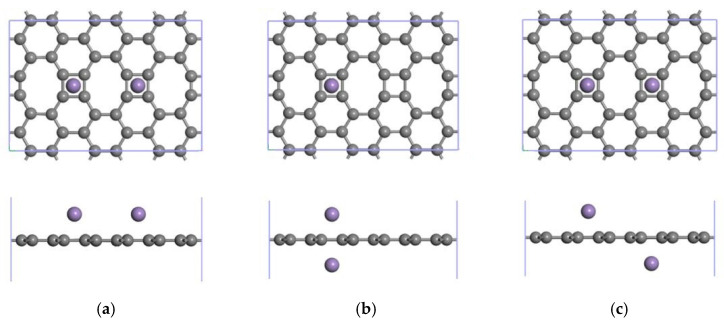
2Mn-ELD-42C graphene configurations (gray balls represent carbon atoms, and purple balls represent Mn atoms). (**a**) The two Mn atoms were located on the same side of the ELD-42C graphene configuration; (**b**) the two Mn atoms were placed on either side of the same quadrilateral carbon ring of the ELD-42C graphene configuration; (**c**) the two Mn atoms were placed on either side of two quadrilateral carbon rings separated by an octagonal carbon ring.

To further study the effect of Mn modification on the CH_4_ storage properties of the ELD-42C graphene configuration, the ELD-42C graphene configuration was modified by two Mn atoms. This enabled three stable structures (Figure 11) to be obtained after optimization, with their structural symmetry being taken into account. In Figure 11a, the two Mn atoms were located on the same side of the ELD-42C graphene configuration. This made it difficult to increase the adsorption capacity for CH_4_ molecules because of the limited adsorption space. As each modifying Mn atom forms an active adsorption site, the adsorption capacity for CH_4_ can be increased more effectively by placing the two Mn atoms on either side of the ELD-42C graphene configuration. Figure 11b shows the two Mn atoms placed on either side of the same quadrilateral carbon ring of the ELD-42C graphene configuration, with an average binding energy of −2.816 eV. In Figure 11c, the two Mn atoms were placed on either side of two quadrilateral carbon rings separated by an octagonal carbon ring, with an average binding energy of −3.451 eV. This was slightly lower than the graphene configuration shown in Figure 11a (−3.556 eV) but produced a more stable structure. In the structure shown in Figure 11c, the binding energy of the first atom (−3.453 eV) was close to that of the second Mn atom (−3.449 eV), proving that the interaction between the two Mn atoms differed to a lesser degree. On the basis of this analysis, the 2Mn-ELD-42C graphene configuration shown in Figure 11c was selected for the study of its CH_4_ adsorption properties.

#### 3.3.2. Adsorption of CH_4_ by the 2Mn-ELD-42C Graphene Configuration

**Figure 12:**
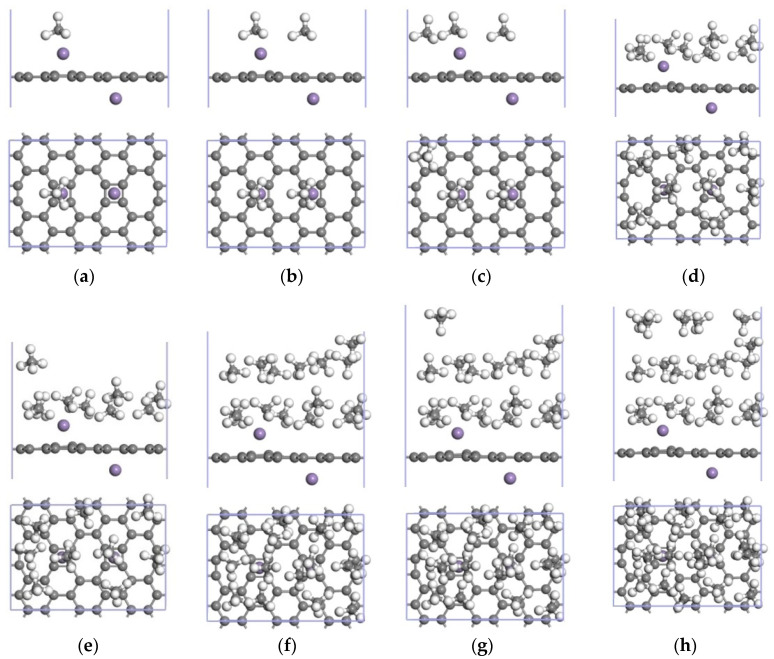
jCH_4_↔2Mn-ELD-42C adsorption configurations (*j* = 1, 2, …, 20). (**a**–**h**) respectively show the 2Mn-ELD-42C graphene configuration with 1–20 CH_4_ molecule(s) adsorbed on one side.

Twenty CH_4_ molecules at most could be stably adsorbed on one side of the 2Mn-ELD-42C graphene configuration. The jCH_4_↔2Mn-ELD-42C adsorption configuration (*j* = 1, 2, …, 20) after optimization is shown in Figure 12. When the ninth CH_4_ molecule was adsorbed by the 2Mn-ELD-42C graphene configuration, stratification occurred due to the repulsion between the positively charged surfaces of the CH_4_ molecules and the limited adsorption space near the Mn atoms (Figure 7e). In other words, the preceding eight CH_4_ molecules were adsorbed in the first layer (Figure 7a–d), near the Mn atoms. This was similar to the monolayer gas adsorption by the Mn-ELD-42C graphene configuration modified by a single Mn atom. When the second Mn atom was added, the increased CH_4_ adsorption sites made the interaction between the Mn atoms and CH_4_ molecules exceed the mutual repulsion between the CH_4_ molecules, so five CH_4_ molecules were able to be adsorbed in the third layer. As noted above, the 2Mn-ELD-42C graphene configuration could stably adsorb twenty CH_4_ molecules at most on a single side, with the adsorption substrate of the optimized 20CH_4_↔2Mn-ELD-42C adsorption configuration forming an arc (Figure 7h). Clearly, the 2Mn-ELD-42C graphene configuration obtained after the second Mn atom was added improved the CH_4_ adsorption capacity of the substrate and increased the number of adsorption sites, effectively remedying the issue with the Mn-ELD-42C graphene configuration when adsorbing CH_4_ molecules at a greater distance from the Mn atoms.

Table 5 gives the average adsorption energy, $\bar{E_{ad}}$, and the adsorption energy, $E_{ad}$, of the jCH_4_↔2Mn-ELD-42C adsorption configuration for CH_4_ adsorption on one side and on both sides; the distance, $d_{CH_{4}-S}$, between the CH_4_ molecules and the 2Mn-ELD-42C graphene configuration; the distance, $d_{CH_{4}-Mn}$, between the CH_4_ molecules and Mn atoms; and the CH_4_ adsorption capacity (PBW) of the 2Mn-ELD-42C graphene configuration. It can be seen that the absolute value of the average adsorption energy decreased with an increase in j, the number of adsorbed CH_4_ molecules. Comparing the adsorption energy, $E_{ad}$, of the eight CH_4_ molecules adsorbed on one side and in a single layer, the first and fourth CH_4_ molecules were closer to the Mn atoms and had smaller adsorption distances from the plane of the graphene configuration, so there was a higher adsorption energy. The adsorption distance between the 20th CH_4_ molecule and the 2Mn-ELD-42C graphene configuration was the largest out of the 20 CH_4_ molecules (11.600 Å), with the average adsorption energy, $\bar{E_{ad}}$, and adsorption energy, $E_{ad}$, also being the smallest (−0.868 eV/CH_4_ and −0.726 eV, respectively). Layered adsorption occurred when the CH_4_ molecules were adsorbed by the 2Mn-ELD-42C graphene configuration, causing the 20th CH_4_ molecule to be the farthest away from Mn atoms; there was repulsion between the positively charged surfaces of the CH_4_ molecules, making the adsorption energy the lowest. When a 21st CH_4_ molecule was placed on one side of the configuration, the calculated adsorption energy became positive, indicating that the gas molecule had not been adsorbed.

**Figure 13:**
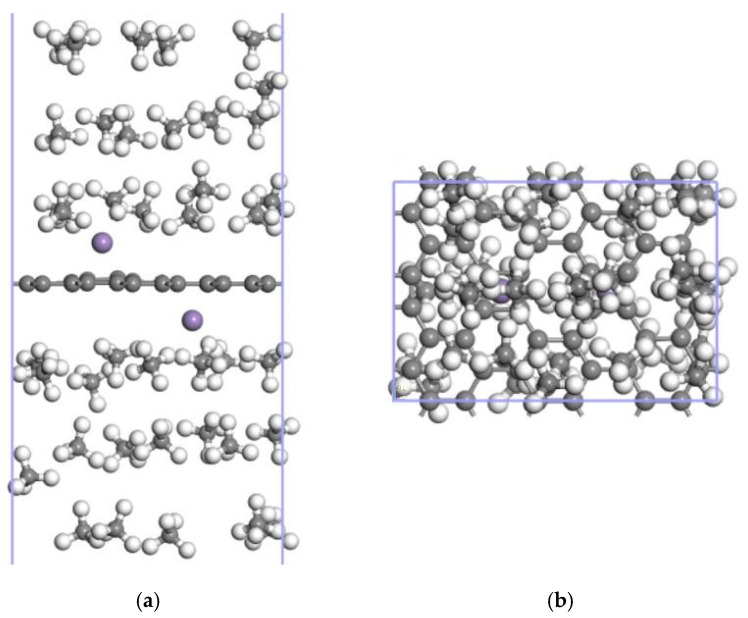
20CH_4_↔2Mn-ELD-42C↔20CH_4_ adsorption configuration with 20 CH_4_ molecules being adsorbed on each side. (**a**) Front view; (**b**) top view (the gray balls represent carbon atoms, the white balls are hydrogen atoms and the purple balls represent Mn atoms).

Up to 40 CH_4_ molecules could be stably adsorbed on both sides of the 2Mn-ELD-42C graphene configuration (see Figure 13), with an average adsorption energy of −0.862 eV/CH_4_ and an adsorption capacity of 51.09 wt%. Note that, although the average adsorption energy of the 2Mn-ELD-42C graphene configuration (−0.862 eV/CH_4_) was close to that of the Mn-ELD-42C graphene configuration (−0.867 eV/CH_4_), the CH_4_ adsorption capacity of the 2Mn-ELD-42C graphene configuration (51.09 wt%) was 1.11 times that of the Mn-ELD-42C graphene configuration.

Although the CH_4_ adsorption capacity of the 2Mn-ELD-42C graphene configuration is lower than some references, as tabulated in Table 6, it goes beyond the specified DOE standard (50 wt%), proving that 2Mn-ELD-42C graphene configurations may offer good opportunities for industrial development and are worth further research and development in the future.

## 4. Conclusions

This paper explored the CH_4_ adsorption properties of ELD-42C, Mn-ELD-42C and 2Mn-ELD-42C graphene configurations using DFT to work from first principles. The study results showed that the CH_4_ adsorption of a basic ELD-42C graphene configuration is weak. Here, the average adsorption energy was −0.842 eV/CH_4_, and the CH_4_ adsorption capacity was 45.26 wt%. Sixteen CH_4_ molecules could be stably adsorbed on the Mn-doped side of the Mn-ELD-42C graphene configuration, and fourteen on the other side, with an average adsorption energy of −0.867 eV/CH_4_ and an adsorption capacity of 46.25 wt%. Thus, the CH_4_ adsorption capacity of Mn-ELD-42C graphene configurations can be effectively enhanced by modification with a single Mn atom. This configuration further improved the CH_4_ adsorption and increased the number of adsorption sites, with it being able to stably adsorb up to 40 CH_4_ molecules across the two sides, with an average adsorption energy of −0.862 eV/CH_4_ and an adsorption capacity of 51.09 wt%. The adsorption capacity of the 2Mn-ELD-42C graphene configuration was 1.13 times that of the ELD-42C graphene configuration and exceeded the proposed DOE standards (50 wt%). Together, these results indicate that 2Mn-ELD-42C graphene configurations have great potential for the development of industrially viable CH_4_ storage materials. The DFT calculation results in this paper only illustrate the CH_4_ adsorption properties of ELD graphene configurations when modified by a single Mn atom or two Mn atoms. In subsequent studies, the effects of environmental factors such as temperature and pressure on the CH_4_ adsorption properties of ELD-42C, Mn-ELD-42C and 2Mn-ELD-42C graphene configurations will be further investigated using molecular dynamics methods.

## Figures and Tables

**Table 1:** Adsorption energy, $E_{ad}$, of the CH_4_↔ELD-42C graphene adsorption configuration; vertical distance, d, between the C atoms in the CH_4_ molecules and the plane of the ELD-42C graphene configuration; and bond angle, ∠H-C-H, between the C atoms and H atoms in the CH_4_ molecules.

The Absorption Point of CH_4_	$E_{ad}\;(\mathrm{eV})$	d (Å)	∠H-C-H (°)
H1	−0.824	3.291	109.507
H2	−0.833	3.222	109.878
H3	−0.847	3.081	109.551
T1	−0.835	3.278	109.554
B1	−0.832	3.251	109.811
B2	−0.830	3.251	109.560

**Table 2:** Adsorption characteristics of a single Mn atom at H1, H2 and H3 on Mn-ELD-42C graphene configurations.

The Absorption Point of a Single Mn Atom	$E_{b_{Mn}}\;(eV)$	Distance (Å)				Δρ (e)
		BL1	BL2	BL3	BL4	
H1	−2.922	2.037	2.037	2.104	2.105	0.29
H2	−3.453	2.967	2.974	2.974	2.981	0.39
H3	−3.218	2.113	2.128	2.430	2.431	0.33

**Table 3:** Adsorption energy and related parameters for CH_4_ molecules in the Mn-ELD-42C graphene configuration.

Number of CH_4_ Molecules	$\bar{E_{ad}}\;(\mathrm{eV}/{\mathrm{CH}}_{4})$	$E_{ad}\;(\mathrm{eV})$	$d_{CH_{4}-S}$ (Å)	$d_{CH_{4}-Mn}$ (Å)	PBW (wt%)
1	−1.717	−1.717	3.824	1.967	2.79
2	−1.289	−0.862	3.789	3.614	5.43
3	−1.177	−0.953	3.590	4.483	7.92
4	−1.103	−0.879	4.100	4.956	10.29
5	−1.047	−0.824	3.551	7.299	12.54
6	−1.022	−0.897	3.448	7.293	14.68
7	−1.018	−0.978	3.397	3.928	16.72
8	−0.995	−0.832	4.840	5.319	18.66
9	−0.971	−0.775	7.176	5.724	20.52
10	−0.956	−0.825	7.156	5.777	22.29
11	−0.943	−0.810	7.329	5.612	23.98
12	−0.927	−0.755	7.460	8.228	25.60
13	−0.918	−0.806	7.831	9.377	27.16
14	−0.913	−0.856	8.035	7.837	28.65
15	−0.907	−0.816	9.132	7.743	30.08
16	−0.897	−0.755	11.550	10.780	31.46
30	−0.867				46.25

**Table 4:** Mulliken layout of the Mn-ELD-42C graphene configuration before and after adsorbing a single CH_4_ molecule.

	Before Adsorption (e)				After Adsorption (e)			
Atom	s	*p*	d	Charge	s	*p*	d	Charge
H_1_	0.73			0.27	0.87			0.13
H_2_	0.73			0.27	0.87			0.13
H_3_	0.73			0.27	0.72			0.28
H_4_	0.73			0.27	0.64			0.36
C	1.51	3.59		−1.10	1.49	3.61		−1.09
C1	1.03	3.00		−0.03	1.05	3.03		−0.08
C2	1.03	3.00		−0.04	1.05	3.03		−0.09
C3	1.05	3.03		−0.09	1.05	3.04		−0.10
C4	1.05	3.04		−0.09	1.05	3.04		−0.10
Mn	2.00	6.00	6.21	0.69	2.01	6.00	6.13	0.98

**Table 5:** Adsorption energy and related parameters for CH_4_ molecules in the 2Mn-ELD-42C graphene configuration.

Number of CH_4_ Molecules	$\bar{E_{ad}}\;(\mathrm{eV}/{\mathrm{CH}}_{4})$	$E_{ad}\;(\mathrm{eV})$	$d_{CH_{4}-S}$ (Å)	$d_{CH_{4}-Mn}$ (Å)	PBW (wt%)
1	−1.726	−1.726	3.789	1.943	2.55
2	−1.296	−0.866	3.828	4.045	4.96
3	−1.149	−0.855	3.590	4.406	7.27
4	−1.105	−0.974	3.483	3.996	9.46
5	−1.058	−0.869	4.196	7.647	11.55
6	−1.030	−0.891	3.937	4.558	13.55
7	−1.015	−0.926	3.379	7.596	15.46
8	−0.993	−0.835	4.498	5.836	17.28
9	−0.967	−0.760	7.187	5.871	19.03
10	−0.950	−0.796	7.167	5.827	20.71
11	−0.936	−0.797	7.385	6.282	22.32
12	−0.926	−0.814	7.574	6.176	23.86
13	−0.917	−0.806	7.885	9.321	25.35
14	−0.909	−0.813	7.931	8.754	26.77
15	−0.900	−0.777	9.242	11.033	28.15
16	−0.889	−0.727	11.369	9.523	29.47
17	−0.882	−0.756	11.336	9.868	30.75
18	−0.875	−0.769	11.578	10.493	31.98
19	−0.871	−0.787	11.450	10.427	33.16
20	−0.868	−0.726	11.600	11.992	34.31
40	−0.862				51.09

**Table 6:** Comparison of CH_4_ adsorption capacity on various carbonaceous structure.

Adsorption Structure	Modified Elements	Temp; Pressure	PBW (wt%)	Interpretation	Ref.
2Mn-ELD-42C	Mn	-	51.09	ELD: Extended line defect	This work
2Ti-GDY	Ti	-	55.24	GDY: graphdiyne	[44]
CNT-PG	-	298 k; 40 bar	44.70	CNT-PG: carbon nanotube-porous graphene	[52]
SWNT	-	303 k; 3.55 MPa	19.80	SWNT: single-walled carbon nanotubes	[53]
2Ti-GY	Ti	-	48.40	GY: graphyne	[8]
2Mn-GR	Mn	-	32.93	GR: graphene	[6]
2Mn-N-GDY	Mn, N	-	58.50	GDY: graphdiyne	[54]
Ti-GDY	Ti	-	63.54	Co-mixing H_2_ and CH_4_	[55]
GRHA/ACNF	-	298 k; 12 bar	66.40	GRHA: graphene-derived rice husk ashes; ACNF: activated carbonNanofibers	[56]

## Data Availability

The data presented in this study are available on reasonable request from the corresponding author.
